# Supplementary material for: Mechanism of activating mutations and allosteric drug inhibition of the phosphatase SHP2
Source: Nat Commun. 2018 Oct 30;9:4507. doi: 10.1038/s41467-018-06814-w (PMC6207724; doi:10.1038/s41467-018-06814-w)
Supplement: Supplementary file 1 — Supplementary Information [file 41467_2018_6814_MOESM1_ESM.pdf]

Supplementary information

Mechanism of activating mutations and allosteric drug inhibition of the phosphatase SHP2

Pádua et al.

a

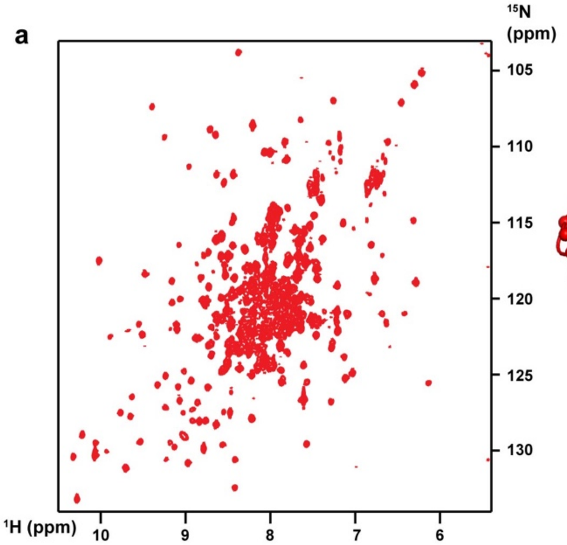

b

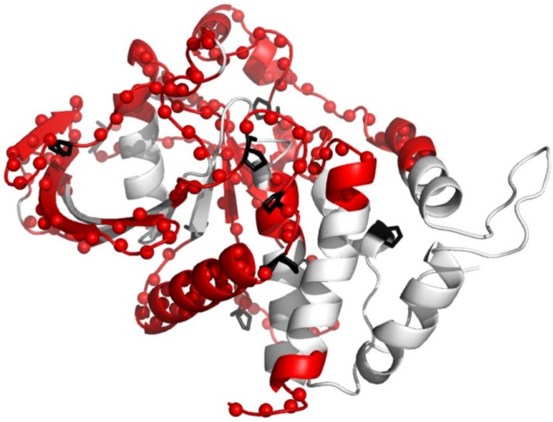

c

```
> PTP
 211 -----LNTTRINAAEIESRVRELSKLAETTDKVKQGFWEEFETLQQQECKLLYSRKEGQRQENKNKNRYK
 281 NILPFDHTRVVLHDGDPNEPVSDYINANIIMPEFETKCNNSKPKKSYIATQGCLQNTVNDFWRMVFQENS
 351 RVIVMTTKEVERGKSKCVKYWPDEYALKEYGVMRVRNVKESAAHDYTLRELKLSKVGQGNTERTVWQYHF
 421 RTWPDHGVPSDPGGVLDFLEEVHHKQESIMDAGPVVVHCSAGIGRTGTFIVIDILIDIIREKGVDCDIDV
 491 PKTIQMVRSQRSGMVQTEAQYRFIYMAVQHYIETLQRRI
```

d

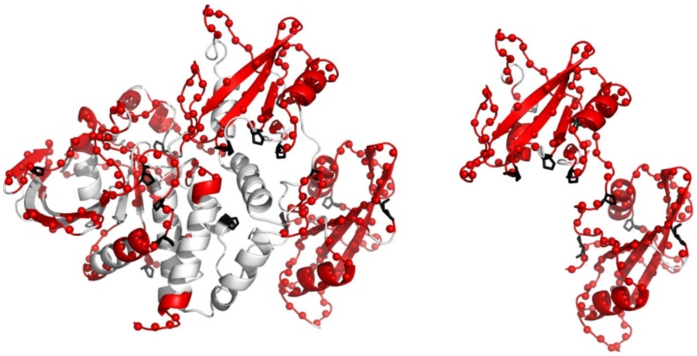

e

```
> FL-WT
   1 MTSRRWFHPNITGVEAENLLLTRGVDGSFLARPSKSNPGDFTLSVRRNGAVTHIKIQNTGDYYDLYGGEK
  71 FATLAELVQYYMEHHGQLKEKNGDVIELKYPLNCADPTSERWFHGHLSGKEAEKLLTEKGKHGSFLVRES
 141 QSHPGDFVLSVRTGDDKGESNDGKSKVTHVMIRCQELKYDVGGGERFDSLTDLVEHYKKNPMVETLGTVL
 211 QLKQPLNTTRINAAEIESRVRELSKLAETTDKVKQGFWEEFETLQQQECKLLYSRKEGQRQENKNKNRYK
 281 NILPFDHTRVVLHDGDPNEPVSDYINANIIMPEFETKCNNSKPKKSYIATQGCLQNTVNDFWRMVFQENS
 351 RVIVMTTKEVERGKSKCVKYWPDEYALKEYGVMRVRNVKESAAHDYTLRELKLSKVGQGNTERTVWQYHF
 421 RTWPDHGVPSDPGGVLDFLEEVHHKQESIMDAGPVVVHCSAGIGRTGTFIVIDILIDIIREKGVDCDIDV
 491 PKTIQMVRSQRSGMVQTEAQYRFIYMAVQHYIETLQRRI
> tandem-SH2-E76K
   1 MTSRRWFHPNITGVEAENLLLTRGVDGSFLARPSKSNPGDFTLSVRRNGAVTHIKIQNTGDYYDLYGGEK
  71 FATLAKLVQYYMEHHGQLKEKNGDVIELKYPLNCADPTSERWFHGHLSGKEAEKLLTEKGKHGSFLVRES
 141 QSHPGDFVLSVRTGDDKGESNDGKSKVTHVMIRCQELKYDVGGGERFDSLTDLVEHYKKNPMVETLGTVL
 211 QLKQPLN
```

Supplementary Figure 1: Backbone assignment of the PTP domain, FL-WT and tandem-SH2-E76K. a) The 2D [^{1}H-^{15}N]-TROSY-HSQC spectrum of PTP at 25 °C. b) The amide nitrogen atoms of the assigned residues are plotted onto the structure of the catalytic domain in red and displayed as spheres. Proline residues are shown in black. c) The assigned residues are colored in red in the amino acid sequence of the PTP construct. d) The amide nitrogen atoms of the assigned residues are plotted onto the structure of the FL-WT and the tandem-SH2 domain in red and displayed as spheres. Proline residues are shown in black. e) The assigned residues are colored in red in the amino acid sequence of the FL-WT and the tandem-SH2 construct.

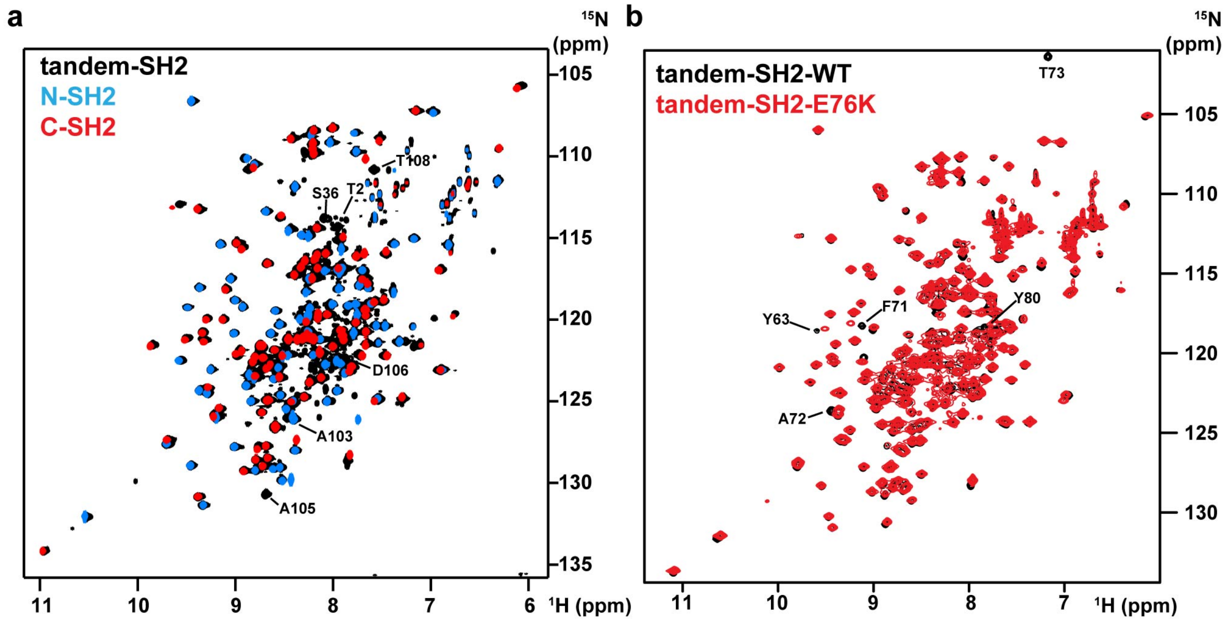

Supplementary Figure 2: Overlay of [^{1}H-^{15}N]-TROSY-HSQC spectra of wild-type and E76K tandem-SH2 and individual SH2 domains.

a) Nearly all cross peaks in the tandem-SH2 (black) domain superimpose with their counterparts in the separate N-SH2 (blue) and C-SH2 (red) domains (data recorded at 35 °C). Differences are only observed for residues in the linker area and/or interface between the two SH2 domains (see the peaks labeled with their assignment). This substantiates the notion that in the context of the tandem-SH2 construct both domains behave as independent entities. b) The [^{1}H-^{15}N]-TROSY-HSQC spectra recorded at 25 °C of tandem-SH2-WT and tandem-SH2-E76K are virtual identical, with minor changes only observed near the site of mutation (see the peaks labeled with their assignment). As a consequence, it is valid to compare the spectra of FL-WT and FL-E76K in Figure 1c to only the tandem-SH2-WT.

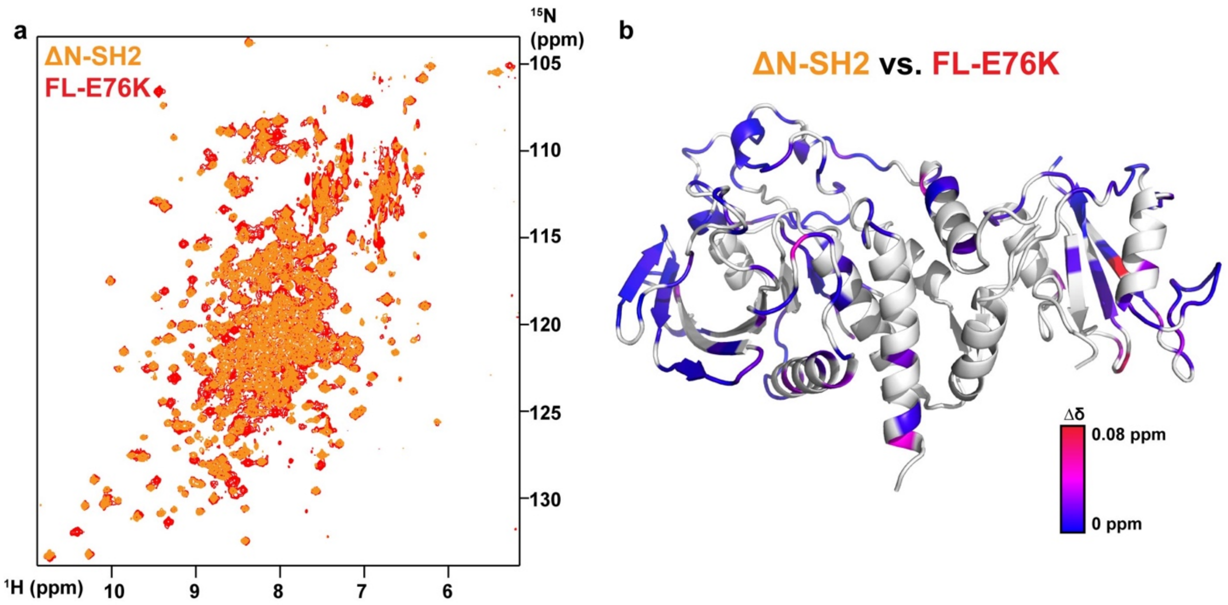

Supplementary Figure 3: Chemical shift differences between ΔN-SH2 and FL-E76K.

a) Superposition of 2D [^{1}H-^{15}N]-TROSY-HSQC spectra of ΔN-SH2 and FL-E76K recorded at 25 °C. b) Chemical shift differences of a) were plotted on the ΔN-SH2 structure (grey indicate unassigned, overlapping, or prolines residues).

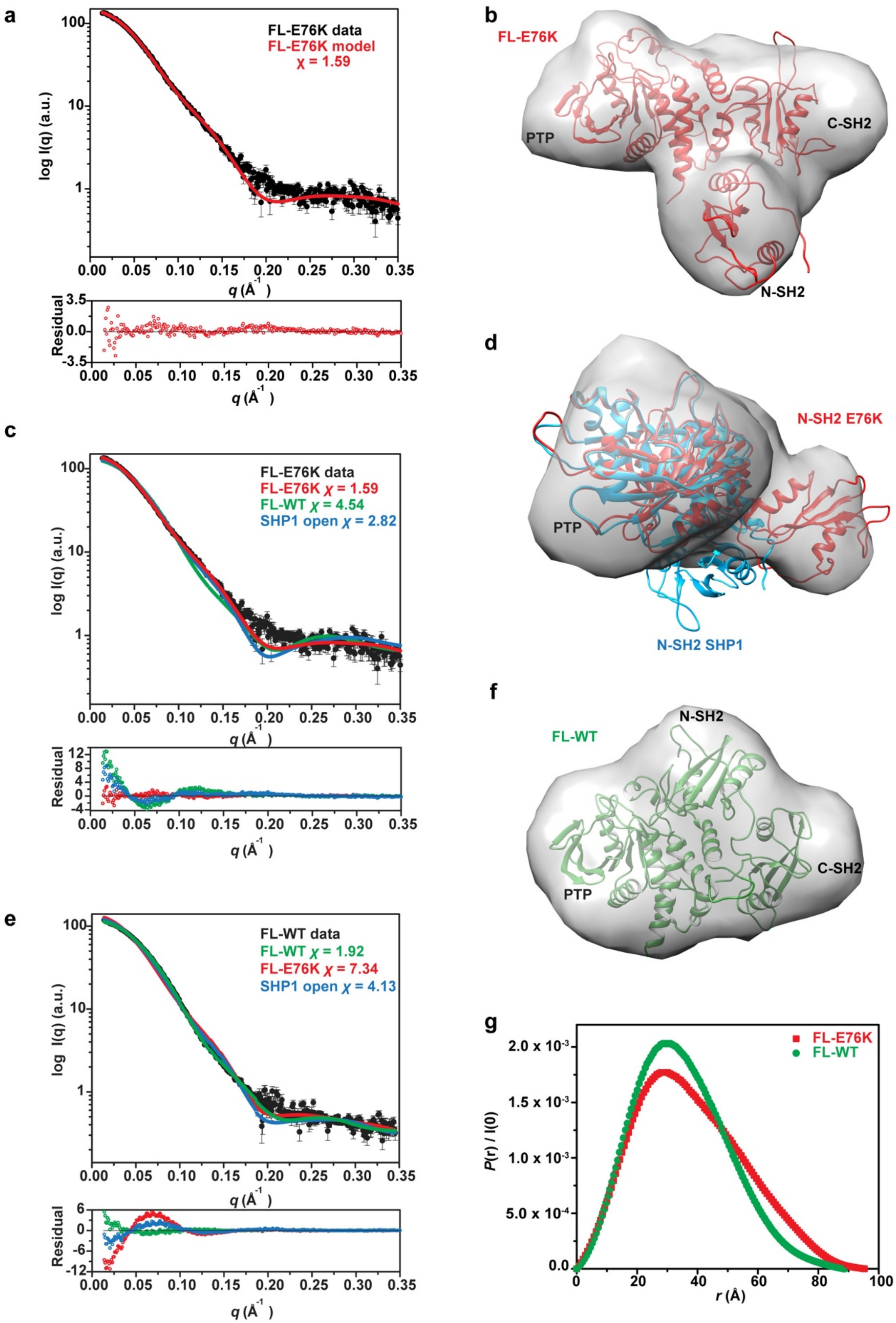
a
FL-E76K data
FL-E76K model
χ = 1.59
log I(q) (a.u.)
q (Å-1)
Residual
b
FL-E76K
PTP
C-SH2
N-SH2
c
FL-E76K data
FL-E76K χ = 1.59
FL-WT χ = 4.54
SHP1 open χ = 2.82
d
N-SH2 E76K
PTP
N-SH2 SHP1
e
FL-WT data
FL-WT χ = 1.92
FL-E76K χ = 7.34
SHP1 open χ = 4.13
f
FL-WT
N-SH2
C-SH2
PTP
g
FL-E76K
FL-WT
P(r) / I(0)
r (Å)

Supplementary Figure 4: Small angle X-ray scattering analyses to investigate the structure of FL-E76K in the open state.

a) Experimental SAXS profile of FL-E76K (black spheres) overlaid with the calculated profile of the best matching open structure of FL-E76K (red, $\chi = 1.59$) and residual plot of the fit to the data at the bottom. b) FL-E76K envelope calculated *ab initio* from SAXS profile showing the N-SH2 protruding out of the PTP. c) Structural model for FL-E76K (red), the crystal structure of FL-WT with modelled loops (green) and the crystal structure of SHP1 (blue) were fit to the SAXS profile of FL-E76K profile ($\chi = 1.59$, 4.54, and 2.82, respectively). d) Crystal structure of SHP1 in the open state (PDB 3ps5)[1] superimposed with FL-E76K/envelope using the PTP domain as reference. The N-SH2 of SHP1 contacts the PTP domain whereas N-SH2 of FL-E76K is detached. e) SAXS data obtained for FL-WT (black spheres) overlaid with calculated profile for FL-WT, FL-E76K, and SHP1 in the open state ($\chi = 1.92$, 7.34 and 4.13, respectively). f) FL-WT envelope calculated *ab initio* from SAXS data and fit to the FL-WT crystal structure. g) Pair distance distribution calculated from FL-WT (green) and FL-E76K (red) SAXS data and normalized to the same area. FL-WT shows a smaller maximum interatomic distance (D_{max} = 88.7Å) when compared to FL-E76K (D_{max} = 95.3 Å).

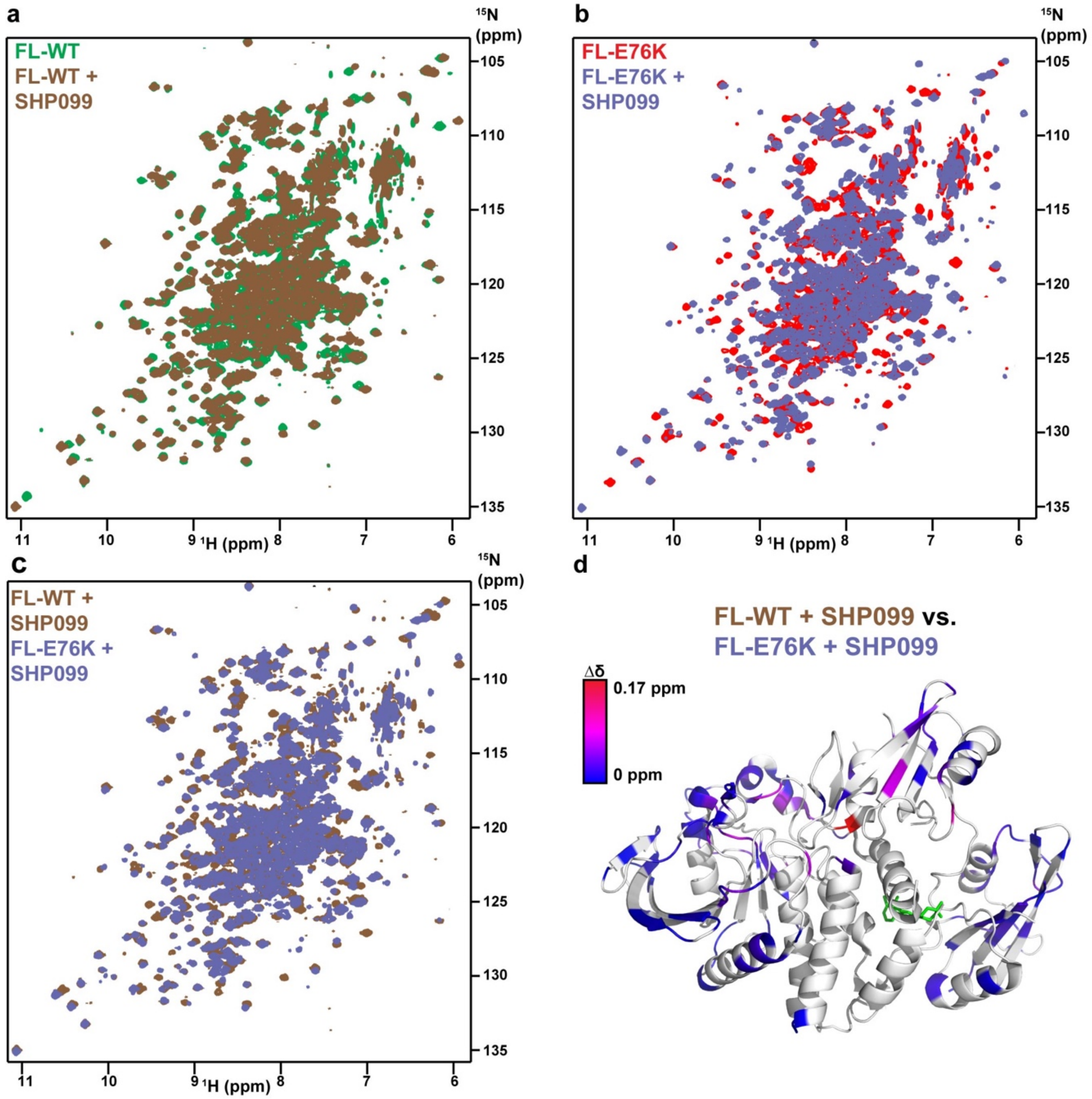

Supplementary Figure 5: Comparison of the [^{1}H-^{15}N]-TROSY-HSQC spectra of FL-WT and FL-E76K in the absence or presence of SHP099 at 35 °C.

a, b) Overlay of [^{1}H-^{15}N]-TROSY-HSQC spectra of SHP2 with/without inhibitor for wild-type (a) and E76K (b) shows larger chemical shift changes for the mutant. This observation is consistent with the notion that FL-WT is already primarily closed, whereas FL-E76K is mainly open. c) Superposition of 2D [^{1}H-^{15}N]-TROSY-HSQC spectra of FL-WT and FL-E76K saturated with SHP099 show remarkable similarities. d) Chemical shift differences of c) were plotted on the closed, inactive crystal structure of FL-WT (PDB 4dgp)[2] and reveal that small changes are observed only along the N-SH2/PTP interface.

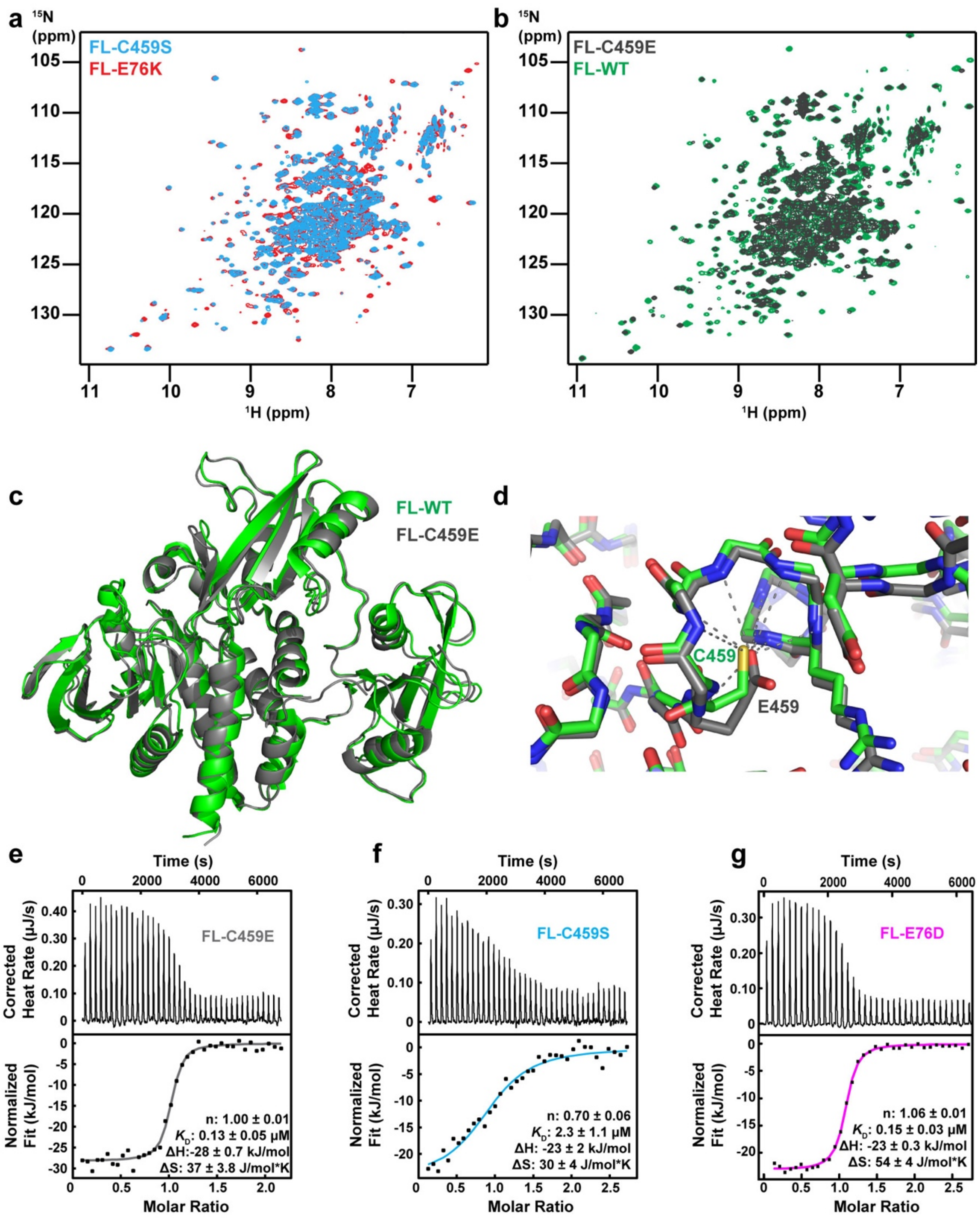

Supplementary Figure 6: NMR spectra and crystallographic data for SHP2 dead mutants and ITC profiles for binding of SHP099 to SHP2 variants.

a,b) Superposition of [^{1}H-^{15}N]-TROSY-HSQC spectra of FL-C459S and FL-E76K (a) and FL-C459E and FL-WT (b) recorded at 35 °C. c) Superposition of the FL-C459E structure (grey) and FL-WT (green)

structure in the closed state. d) The thiolate negative charge in the FL-WT (green) is stabilized by the backbone amides and the N-terminus α-helix dipole. To maintain this charge distribution in an inactive SHP2 mutant, the catalytic cysteine was mutated to a glutamic acid (FL-C459E, grey). Dashed lines correspond to distances within a 3.4 - 5.2 Å range. e) 35.2 μM of FL-C459E titrated with 300 μM of SHP099. f) 26.3 μM of FL-C459S titrated with 300 μM of SHP099. g) 25.3 μM of FL-E76D titrated with 300 μM of SHP099.

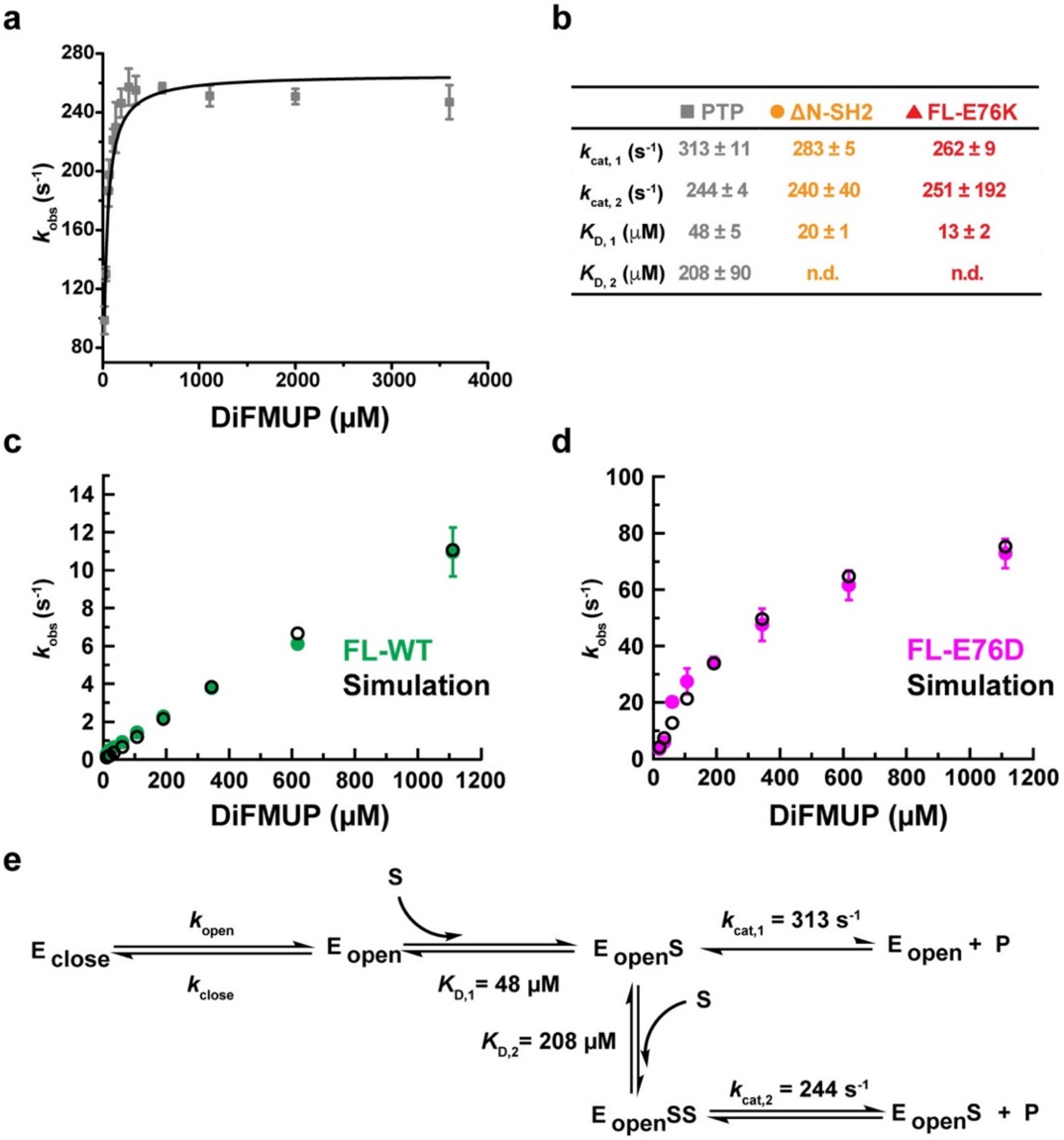

Supplementary Figure 7: Analysis of steady-state kinetics of phosphatase activity.

a) Phosphatase activity for PTP (grey) cannot be explained by a simple Michaelis-Menten model (black). b) A modified Michaelis-Menten model accounting for partial substrate inhibition was used to fit the data and the determined parameters are given. Our analysis shows that $K_{D,2}$ for ΔN-SH2 and FL-E76K is at least an order of magnitude weaker than $K_{D,1}$; however, the exact value is not well constrained by our data and, therefore, not reported. c-d) Using the populations extracted from chemical shift analysis and the steady-state parameters from the PTP domain (see Methods and panel b), the k_{obs} for FL-WT (c) and FL-E76D (d) was simulated using the final kinetic model shown in (e). While marginal differences are observed, the

remarkable correlation between turnover rates and simulated values confirms that SHP2 activity is primarily regulated by the equilibrium between closed, inactive and open, active species. Data points and error bars in a are the average and standard deviation of the analysis of replicate experiments (n=3).

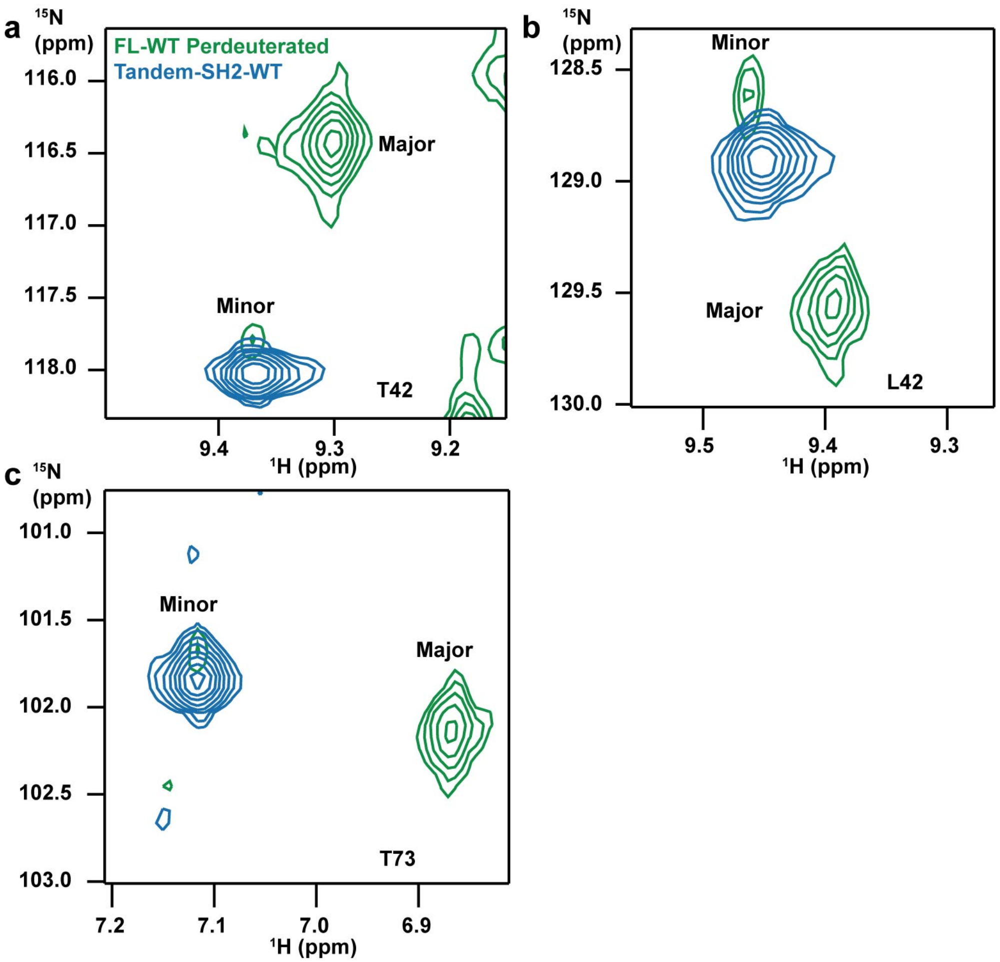

Supplementary Figure 8: Direct observation of FL-WT sampling the open state. (a-c) Representative spectral overlays for residues in the N-SH2 with observed peak duplication. For protonated samples, only the major state is observable and the minor state is broadened beyond detection likely due to the dipolar interaction. In contrast, a long [^{1}H-^{15}N]-TROSY-HSQC of perdeuterated FL-WT shows peak duplication with minor peaks appearing at positions of the analogous tandem-SH2-WT cross peaks. In the perdeuterated sample, the nitrogen peak position is shifted with respect to cross peaks in the protonated tandem-SH2-WT due to the isotope effect.

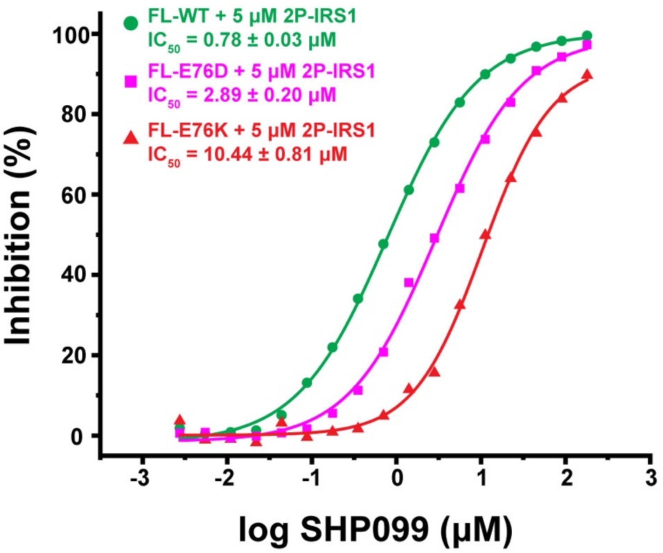

Supplementary Figure 9: Inhibition constants for SHP099 to FL-WT, FL-E76K, and FL-E76D in the presence of 5 μM synthetic, bisphosphorylated IRS-1 peptide. As expected, the observed IC_{50} values are higher in the presence of the activating peptide due to competition: the peptide shifts SHP2 towards the open conformation, whereas the drug can only bind to the closed state. Nevertheless, the trend for the IC_{50} values with respect to wild-type SHP2 and mutant forms remains the same (*cf.* Fig. 6g.).

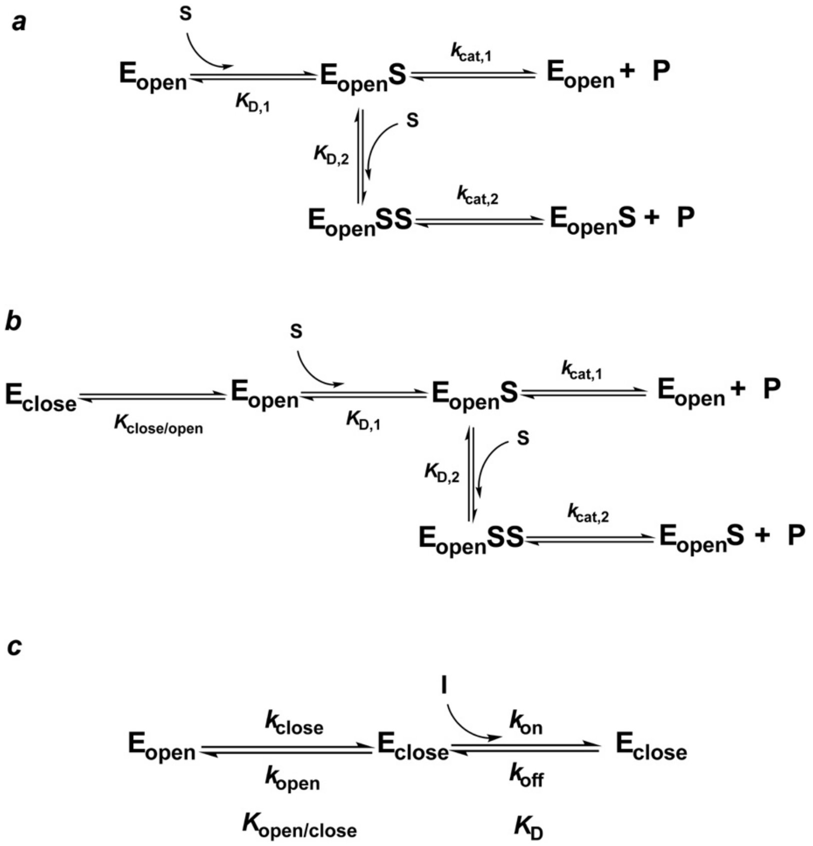

Supplementary Figure 10: SHP2 reaction schemes. a) The modified Adair-Pauling model describing two sequential substrate binding events was used to fit steady-state kinetics of PTP, FL-E76K and ΔN-SH2 constructs. b) An extended model, with the addition of a conformational selection step in the ligand-free protein, was used to simulate the FL-WT and FL-E76D steady-state kinetics. c) Scheme for the conformational selection mechanism used in the analysis of SHP099 binding kinetics.

Supplementary Table 1. Codon optimized FL-WT SHP2 sequence for *E. coli* expression

pET-28a(+) FL-WT SHP2 (1-529) cloned between *Nde*I and *Nhe*I restriction sites
catatggaaaatctgtattttcagggtagcggcatgacctctcgtcgctggttccatccgaatattacgggtgttgaagcggaaaacctgctgctgaccc<br>gtggtgtcgatggctcatttctggcccgcccgagcaaatctaacccgggtgacttcaccctgtcggttcgtcgcaatggcgcagtcacccacattaaa<br>atccagaacacgggcgactattacgatctgtacggcggtgaaaaatttgcgaccctggccgaactggttcaatattacatggaacatcacggtcagct<br>gaaagagaaaaacggcgatgtgatcgaactgaaatatccgctgaactgcgcagacccgacgtcagaacgttggttccatggtcacctgtcgggcaa<br>agaagctgaaaaactgctgaccgaaaaaggtaaacatggcagctttctggtgcgtgaaagtcagtcccacccgggtgatttcgttctgtctgtccgca<br>cgggtgatgacaaaggcgaatcaaatgacggcaaatcgaaagtgacccatgttatgattcgttgtcaggaactgaaatacgatgttggcggtggcga<br>acgctttgacagcctgaccgatctggtggaacactataagaaaaacccgatggtggaaaccctgggtacggttctgcagctgaaacaaccgctgaat<br>accacgcgcattaacgcggccgaaatcgaaagtcgtgttcgcgaactgtccaaactggccgaaaccacggataaagtgaaacagggtttttgggaa<br>gaattcgaaaccctgcagcaacaggaatgcaaactgctgtacagtcgtaaagaaggccagcgccaagaaaacaaaaacaaaaaccgttacaaaaa<br>catcctgccgttcgatcatacgcgcgtggttctgcacgatggtgacccgaacgaaccggtgtctgactacatcaatgcgaacattatcatgccggaatt<br>cgaaaccaaatgcaacaacagcaaaccgaaaaaatcttacatcgccacccagggctgtctgcaaaatacggttaacgatttttggcgtatggtcttcca<br>ggaaaatagccgcgtcattgtgatgaccacgaaagaagtggaacgtggtaaatctaaatgtgttaaatactggccggatgaatacgcactgaaagaat<br>atggcgtcatgcgtgtgcgcaatgttaaagaaagtgcagctcatgattacaccctgcgcgaactgaaactgtccaaagttggtcagggcaacaccga<br>acgtacggtgtggcagtatcattttcgtacctggccggaccatggtgtgccgagcgatccgggtggcgttctggacttcctggaagaagtccatcaca<br>aacaggaaagcattatggatgcaggtccggtcgtggttcattgctctgctggtatcggccgtaccggcacgttcatcgttatcgacatcctgatcgatat<br>catccgcgaaaaaggtgtcgattgtgacattgatgtgccgaaaacgatccagatggtccgttcacaacgctcgggcatggtgcagaccgaagcgca<br>atatcgtttcatctacatggccgtgcagcattatattgaaaccctgcaacgtcgcatctgagctagc

Supplementary Table 2. Codon optimized ΔN-SH2 SHP2 sequence for *E. coli* expression

pET-28a(+) ΔN-SH2 SHP2 (104-529) cloned between *Nde*I and *Nhe*I restriction sites
catatggaaaatctgtattttcagggtagcggctgcgcagacccgacgtcagaacgttggttccatggtcacctgtcgggcaaagaagctgaaaaact<br>gctgaccgaaaaaggtaaacatggcagctttctggtgcgtgaaagtcagtcccacccgggtgatttcgttctgtctgtccgcacgggtgatgacaaag<br>gcgaatcaaatgacggcaaatcgaaagtgacccatgttatgattcgttgtcaggaactgaaatacgatgttggcggtggcgaacgctttgacagcctg<br>accgatctggtggaacactataagaaaaacccgatggtggaaaccctgggtacggttctgcagctgaaacaaccgctgaataccacgcgcattaac<br>gcggccgaaatcgaaagtcgtgttcgcgaactgtccaaactggccgaaaccacggataaagtgaaacagggtttttgggaagaattcgaaaccctg<br>cagcaacaggaatgcaaactgctgtacagtcgtaaagaaggccagcgccaagaaaacaaaaacaaaaaccgttacaaaaacatcctgccgttcgat<br>catacgcgcgtggttctgcacgatggtgacccgaacgaaccggtgtctgactacatcaatgcgaacattatcatgccggaattcgaaaccaaatgcaa<br>caacagcaaaccgaaaaaatcttacatcgccacccagggctgtctgcaaaatacggttaacgatttttggcgtatggtcttccaggaaaatagccgcgt<br>cattgtgatgaccacgaaagaagtggaacgtggtaaatctaaatgtgttaaatactggccggatgaatacgcactgaaagaatatggcgtcatgcgtgt<br>gcgcaatgttaaagaaagtgcagctcatgattacaccctgcgcgaactgaaactgtccaaagttggtcagggcaacaccgaacgtacggtgtggca<br>gtatcattttcgtacctggccggaccatggtgtgccgagcgatccgggtggcgttctggacttcctggaagaagtccatcacaaacaggaaagcatta<br>tggatgcaggtccggtcgtggttcattgctctgctggtatcggccgtaccggcacgttcatcgttatcgacatcctgatcgatatcatccgcgaaaaagg<br>tgtcgattgtgacattgatgtgccgaaaacgatccagatggtccgttcacaacgctcgggcatggtgcagaccgaagcgcaatatcgtttcatctacat<br>ggccgtgcagcattatattgaaaccctgcaacgtcgcatctgagctagc

Supplementary Table 3. Primer sequences

Construct	Forward primer	Reverse primer
FL-E76K	5'-aaaatttgcgaccctggccaaactggttcaatattacatg-3'	5'-catgtaatattgaaccagtttggccagggtcgcaaatttt-3'
FL-E76D	5'-ttgcgaccctggccgatctggttcaatattacat-3'	5'-atgtaatattgaaccagatcggccagggtcgcaa-3'
FL-C459E	5'-tgcaggtccggtcgtggttcatgagtctgctggtatcg-3'	5'-cgataccagcagactcatgaaccacgaccggacctgca-3'
FL-C459S	5'-tccggtcgtggttcatagctctgctggtatc-3	5'-gataccagcagagctatgaaccacgaccgga-3'
ΔN-SH2	5'-gctgaactgcgcagactagacgtcagaacgttgg-3'	5'-ccaacgttctgacgtctagtctgcgcagttcagc-3'
tandem-SH2	5'-gttctgcagctgaaacaaccgctgaattagacgcgcattaacgc-3'	5'-gcgttaatgcgcgtctaattcagcggttgtttcagctgcagaac-3'

Supplementary Table 4. X-ray crystallography data collection and refinement statistics

	FL-C459E PDB 6cmp	ΔN-SH2 PDB 6cmq	FL-E76D + SHP099 PDB 6cmr	FL-E76K + SHP099 PDB 6cms
**Data collection**				
Space group	$P2_12_12_1$	$P2_1$	$P22_12_1$	$P22_12_1$
Cell dimensions				
a, *b*, *c* (Å)	54.75, 84.91, 211.92	69.55, 56.2, 247.46	41.27, 54.36, 221.53	40.72, 54.14, 218.85,
α, β, γ (°)	90, 90, 90	90, 93.99, 90	90, 90, 90	90, 90, 90
Resolution (Å)	52.98 - 1.803 (1.867 - 1.803)	82.29 - 2.9 (3.004 - 2.9)	52.79 - 2.21 (2.289 - 2.21)	54.71 - 2.682 (2.778 - 2.682)
R_{sym} or R_{merge}	0.1599 (2.524)	1.035 (1.548)	0.1645 (1.699)	0.6201 (1.828)
$I / \sigma I$	7.32 (0.61)	4.28 (1.52)	5.85 (0.96)	113.65 (8.13)
Completeness (%)	99.22 (94.05)	98.53 (98.71)	99.92(100.00)	99.23 (92.54)
Redundancy	6.6 (4.8)	4.6 (4.7)	6.1 (6.6)	93.9 (95.6)
**Refinement**				
Resolution (Å)	52.98 - 1.803 (1.867 - 1.803)	82.29 - 2.9 (3.004 - 2.9)	52.79 - 2.21 (2.289 - 2.21)	54.71 - 2.682 (2.778 - 2.682)
No. reflections	91347	42398	25954	14212
R_{work} / R_{free}	0.2017/0.2291	0.2491/0.2758	0.2251/0.2596	0.2047/0.2651
No. atoms	8653	12518	3952	4125
Protein	7889	12516	3850	4035
Ligand/ion			23	23
Water	764	2	79	67
B-factors	31.87	44.78	53.28	51.34
Protein	31.48	44.78	53.56	51.71
Ligand/ion			43.56	39.40
Water	35.91	28.05	42.03	33.08
R.m.s. deviations				
Bond lengths (Å)	0.005	0.003	0.003	0.002
Bond angles (°)	1.02	0.89	0.90	0.48

Data from a single crystal was used to solve each of the following structures: FL-C459E, ΔN-SH2 and FL-E76D + SHP099. Data from two crystals were merged to solve the FL-E76K + SHP099 structure.
*Values in parentheses are for highest-resolution shell.

Supplementary Table 5. SAXS data collection and modelling parameters

**Data collection**		
Instrument	Stanford Synchrotron Radiation Source BL4-2/ Rayonix MX225-HE detector	
Wavelength (Å)	1.127	
Beam size (mm)	300 x 500	
Camera length (m)	1.7	
q measurement range ($Å^{-1}$)	0.0066 - 0.4904	
exposure time	1s per frame/ 10 frames per image	
**Software for data reduction, analysis and interpretation**		
SAXS data reduction	SAXSpipe (available at SSRL BL4-2), SAXSMerge[3]	
Basic analyses: Guinier, P(**r**)	PRIMUSqt from ATSAS 2.8.3[4]	
Shape modelling	DAMMIN[5] via ATSAS online (https://www.embl-hamburg.de/biosaxs/atsas-online/)	
Atomic structure modelling	FoXS[6], MultiFoXS[7]	
Missing sequence modelling	MODELLER v9.20[8], Allosmod[9]	
Three-dimensional graphic model representations	PyMOL[10], Chimera[11]	
**Structural parameters**	Sample	
**Guinier analysis**	FL-WT	FL-E76K
I(0)	121.13 ± 0.25	142.7 ± 0.44
R_g (Å)	26.85 ± 0.43	29.1 ± 0.62
q range for Guinier ($Å^{-1}$)	0.0159-0.0479	0.0150-0.0443
$q\ R_g$ (max)	1.29	1.29
**P(r) analysis**		
I(0)	121.1	143.3
R_g (Å)	26.86	29.46
D_{max} (Å)	88.7	95.3
q range ($Å^{-1}$)	0.016-0.2978	0.0150-0.2740

Supplementary References

1. Wang, W. *et al.* Crystal structure of human protein tyrosine phosphatase SHP-1 in the open conformation. *J Cell Biochem* **112**, 2062-2071, (2011).
2. Yu, Z. H. *et al.* Structural and mechanistic insights into LEOPARD syndrome-associated SHP2 mutations. *J Biol Chem* **288**, 10472-10482, (2013).
3. Spill, Y. G. *et al.* SAXS Merge: an automated statistical method to merge SAXS profiles using Gaussian processes. *J Synchrotron Radiat* **21**, 203-208, (2014).
4. Franke, D. *et al.* ATSAS 2.8: a comprehensive data analysis suite for small-angle scattering from macromolecular solutions. *J Appl Crystallogr* **50**, 1212-1225, (2017).
5. Svergun, D. I. Restoring Low Resolution Structure of Biological Macromolecules from Solution Scattering Using Simulated Annealing. *Biophys. J.* **76**, 2879-2886, (1999).
6. Schneidman-Duhovny, D., Hammel, M., Tainer, J. A. & Sali, A. Accurate SAXS profile computation and its assessment by contrast variation experiments. *Biophys J* **105**, 962-974, (2013).
7. Schneidman-Duhovny, D., Hammel, M., Tainer, J. A. & Sali, A. FoXS, FoXSDock and MultiFoXS: Single-state and multi-state structural modeling of proteins and their complexes based on SAXS profiles. *Nucleic Acids Res* **44**, W424-W429, (2016).
8. Sali, A. & Blundell, T. L. Comparative protein modelling by satisfaction of spatial restraints. *J Mol Biol* **234**, 779-815, (1993).
9. Weinkam, P., Pons, J. & Sali, A. Structure-based model of allostery predicts coupling between distant sites. *Proc Natl Acad Sci U S A* **109**, 4875-4880, (2012).
10. PyMOL. The PyMOL Molecular Graphics System v. 1.8 (Schrödinger, New York, 2017).
11. Pettersen, E. F. *et al.* UCSF Chimera--a visualization system for exploratory research and analysis. *J Comput Chem* **25**, 1605-1612, (2004).
